# Non-cardiovascular findings on chest CT angiography in children with congenital heart disease: How important are they?

**DOI:** 10.1186/s12880-022-00739-z

**Published:** 2022-01-22

**Authors:** Yaotse Elikplim Nordjoe, Suzanne Rita Aubin Igombe, Latifa Chat

**Affiliations:** Pediatric Radiology Department, Children’s Hospital, Centre Hospitalo-Universiataire Ibn Sina, Rue Lamfadel Cherkaoui Rabat - Institut, B.P 6527, Rabat, Morocco

**Keywords:** Congenital heart disease, Chest CT angiography, Children, Non-cardiovascular findings

## Abstract

**Background:**

There are only a few publications about the non-cardiovascular findings in children with congenital heart diseases explored by chest CT angiography. The purpose of our study is to evaluate the prevalence of non-cardiovascular findings on chest CT angiographies in children with congenital heart disease and to raise awareness about their importance among the radiologists.

**Methods:**

We retrospectively reviewed the 272 chest CT angiographies performed in our pediatric radiology department between January 2017 and march 2021 and extracted the data of the 180 patients positive for a congenital heart disease. Then from that pool, we sorted out the non-cardiovascular findings into significant and non-significant in regard of their relevance or not in the patient’s management.

**Results:**

Non-cardiovascular lesions were found in 58% (105/180) of our patients, and 49% (88/180) of them presented at least one significant non-cardiac lesion. Lung and airways abnormalities were found in 41% (74/180) of the cases, representing the majority of the non-cardiovascular findings. Syndromic associations were found in 17% (28/180) of our patients, including 14 cases of heterotaxic syndrome.

**Conclusion:**

Non-cardiovascular findings are common in children with congenital heart disease. Reporting these associated lesions is a requisite for an optimal therapeutic management of these children. Radiologists should never forget that a Chest CT angiography in children is first and foremost a chest CT.

## Background

The congenital heart diseases are relatively common and very diverse, with an estimated prevalence of 1–4 per 1000 births [1–3]. In children, in order to align with the ALARA "As Low As Reasonably Achievable" principle, cardiac ultrasound and MRI represent excellent imaging tools for diagnosing and monitoring patients with congenital heart disease [4, 5]. However, they are not perfect and not suitable for all situations.

With the development of multi-detector scanners, dose reduction techniques, better reconstruction algorithms and global advances in the field of computer science, chest CT angiography has become and accepted as a rapid and non-invasive diagnosing and monitoring tool in children with congenital heart disease [4, 6–11].

CT allows a detailed regional analysis and therefore, enables the detection of findings other than cardiovascular. These non-cardiovascular findings can have a major impact in the management of the patient. Their prevalence and implications have been the subject of numerous publications in adult cardiac pathology with a highly variable prevalence ranging from 7.1 to 81% [12–19]. On the contrary, in the pediatric population, there are only three publications studying non-cardiovascular findings in congenital heart disease, two on CT angiography [20, 21] and the other on MR angiography [22].

The aims of our work are to evaluate the prevalence and relevance of non-cardiovascular findings on chest CT angiography in children with congenital heart disease in our institution and to raise awareness among the radiologists about the importance of their detection.

## Material and methods

### Description of the study

This is a retrospective observational study over a period of 4 years and 3 months (between January 2017 and march 2021). Analysis was performed on the database of the radiology department of the children's hospital in Rabat—Morocco. We reviewed all the 272 chest CT angiographies performed in the above-mentioned period, for a known or suspected congenital heart disease, of which the 180 patients positive for a congenital heart disease formed the pool of our study. From that pool, non-cardiovascular findings have been identified and processed to evaluate their prevalence and their relevance.

Patients had to be less than 18 years old.

Since the study is based on a retrospective analyze of our archiving database, the obtention of a written informed consent was waived by our institution ethical committee.

### CT angiography procedure

#### Preparation of the patients

A reassurance of the patient was achieved by the parents and the medical staff. If needed, one of the parents was authorized—after protection—to remain in the machine room with the child. A peripheral intravenous access was obtained using a 20 to 24 Gauge catheter placed in an antecubital forearm or hand vein. A little sedation was sometimes necessary; performed by the anesthesiologist assigned to the scanner.

#### CT examination protocol

All the exams were performed on a single-source 16-channel MDCT scanner (SIEMENS Somatom Emotion 16).

An integrated pre-set chest CT angiography protocol was used, allowing the adaptation of the acquisition parameters to the pediatric population: 80 -100 kV, with for example 30 mAs at 3 kg and 40 mAs at 5 kg. All the examinations were non-electrocardiogram-gated and were acquired during shallow free-breathing or suspended inspiration. Contrast medium was power injected (Medrad; Stellant), using 1–2 ml/kg of nonionic contrast medium (Ultravist 300 mg I/ml; Bayer Schering Pharma) at a rate of 0.5–3.0 ml/s, followed by 10–30 ml of saline at a similar rate. Automated bolus tracking was used to achieve optimal opacification. Data sets were reconstructed into 1 to 1.5 mm contiguous slices. We only used filtered back projection algorithm for the reconstructions. Reconstructed slice thickness was 1.5 mm with H30 kernel for mediastinum and 1 mm with H80 kernel for lung. The mean DLP was 45.56 mGy/cm (± 23.81). The mean CTDIv was 1.57 mGy (± 0.52).

### Definition of non-cardiovascular findings

Non-cardiovascular findings are abnormalities other than those of the heart and vessels, discovered on the volume acquired.

A non-cardiovascular finding was “significant” if further investigations were needed and/or if this abnormality contributed to the worsening of the patient’s clinical state.

A “significant” finding could either be “expected” or “unexpected”. An “expected” lesion is one whose occurrence is not surprising, being part of the usual associated lesions of that particular congenital heart disease while an “unexpected” lesion is just the opposite of that.

It is important to note that the same finding can be significant in one patient but non-significant in another one, depending on its impact on the patient's condition. Also, there are some findings whose categorization cannot be formally certain because the physio-pathological implications are complex.

### Data collection and analysis

Post processing and analysis were carried out on a dedicated post processing workstation with SyngoVia software, by a certified senior radiologist with 10 years of experience in congenital heart diseases (Dr L.C.).

The entire arsenal of this platform was used, allowing both usual multiplanar reformatting and MIP, MInIP, VRT 3D reconstructions etc.

Images and reports were archived on our picture archiving and communication systems VISIONPACS and VISIONRIS.

The retrospective analysis of the data was carried out on these above-mentioned archiving platforms. First, all CT scans performed for the indication of known or suspected congenital heart disease were extracted. A second sorting allowed the separation of the normal examinations from the examinations on which there was indeed a congenital heart disease. Lastly, the pool of congenital heart disease was analyzed to deduce those with associated non-cardiovascular findings. These non-cardiovascular findings were subcategorized into: infectious, malformative, tumoral lesions and others. Also, an anatomical distribution of these abnormalities was made, into supra-diaphragmatic findings (pulmonary and airways, mediastinal) and infra-diaphragmatic findings (liver, kidney, spleen, bowel). Bone skeletal abnormalities have also been isolated and finally, syndromic associations were identified.

## Results

### Overall results

A total of 272 chest CT angiographies was performed for a known or suspected congenital heart disease, of which, 180 was positive for a congenital heart disease. The mean age was 3.1 years (± 3.2), ranging from 1 day to 17 years. Some of our patients (n = 20) had a history of surgery (Table 1).

**Table 1:** Characteristics of the study population

Population with congenital heart disease	Mean age ± SD	Range
n = 180 (99 M / 81F)	3,1 years ± 3,2	1 day–17 years

Patients with or without non-cardiovascular findings	n = patients
Patients with non-cardiovascular finding	105 (58% of 180)
Patient without non-cardiovascular findings	75 (42% of 180)

History of surgical procedure	n = patients
Blalock–Taussig–Thomas	11
Coarctation repair	3
Fontan	1
Pulmonary artery prosthesis	1
Tetralogy of Fallot repair	3
Senning	1

Repartition by congenital heart disease	n = patients
Left obstructive lesions	42
Right obstructive lesions	33
Cono-truncus anomalies	30
Congenital arch anomalies	30
Septation anomalies	35
Pulmonary venous anomalies	10

We detected 155 non-cardiovascular anomalies in 105 patients positive for congenital heart disease (58% of 180), of which 88 patients had significant anomalies and 17 patients had non-significant anomalies. More than one non-cardiovascular anomalies were found in 24 patients (23% of 105). In these cases where there were association of several abnormalities in the same patient, if there is only one significant finding; the patient was classified as significant (Table 2).

**Table 2:** Distribution of significant and non-significant findings

Non-cardiovascular findings	n = Patients (% of 180)
Significant	88 (49%)
Expected	66 (37%)
Unexpected	22 (12%)
Non-significant	17 (9%)
Total	105 (58%)

**Fig. 1:**
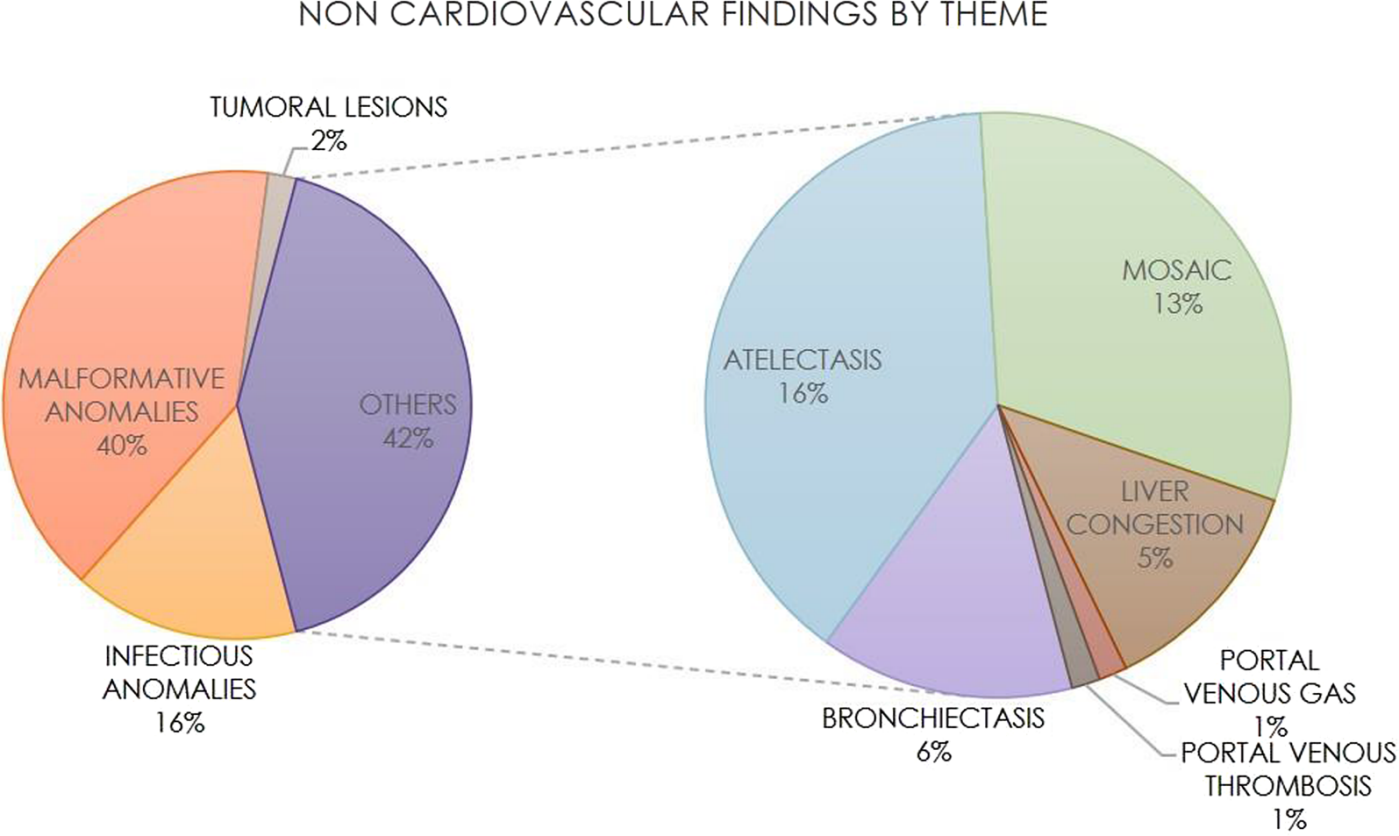
Nature of the non-cardiovascular findings. Distribution of the 155 non-cardiovascular findings detected in the 105 patients positive for congenital heart disease (58% of 180)

Non-cardiovascular findings were classified by subcategories: infectious, malformative, tumoral and others (Fig. 1).

### Topographic distribution and syndromic associations

The non-cardiovascular findings were subdivided into four main groups: supra diaphragmatic, infra diaphragmatic, skeletal and syndromic associations.

#### Supra-diaphragmatic anomalies (Table 3)

Two subsets were isolated. On the one hand lung and airways findings were identified in 74 (41% of the 180 patients of our series) patients and on the other, mediastinal findings were identified in 3 patients. Lung and airways findings were by far the most frequently encountered anomalies, mainly dominated by infectious pneumopathies, mosaic attenuation pattern and atelectasis. Associations can be found in the same patient.

**Table 3:** Distribution of supra-diaphragmatic findings

Localization	Anomalies	n = Patients
Lung and airways	Pulmonary hypoplasia	5
	Pneumonia	23
	Bronchiectasis	9
	Atelectasis	25
	Mosaic attenuation pattern	20
	Emphysema	8
	Pulmonary sequestration	3
	Vascular compression of the trachea	3
Mediastinum	Pericardial effusion	1
	Thymic agenesis	2

#### Infra-diaphragmatic anomalies (Table 4, Figs. 2, 3)

Four subsets were isolated: Liver, kidney, spleen and bowel. Splenic anomalies were essentially asplenia or polysplenia mainly integrated into heterotaxic syndromes. The most frequent liver abnormalities are represented by hepatic congestion, due to hemodynamic changes. Bowel anomalies were essentially malformative with a predominance of hiatal hernias. Kidney anomalies were also malformative, with a horseshoe kidney being the predominant finding.

**Table 4:** Distribution of infra-diaphragmatic and skeletal findings

Organ	Anomaly	n (Patients)
Spleen	Asplenia	10
	Polysplenia	14
Liver	Congestion	8
	Hemangioma	3
	Portal venous thrombosis	1
	Portal venous gas	1
	Simple hepatic cyst	1
Kidney	Horseshoe kidney	4
	Renal agenesis	2
	Dilation of the urinary tract	2
Intestines	Hiatal hernia	3
	Common mesentery	1
	Omphalocele	1
Bone	Scoliosis	3
	Vertebral malformation	2

**Fig. 2:**
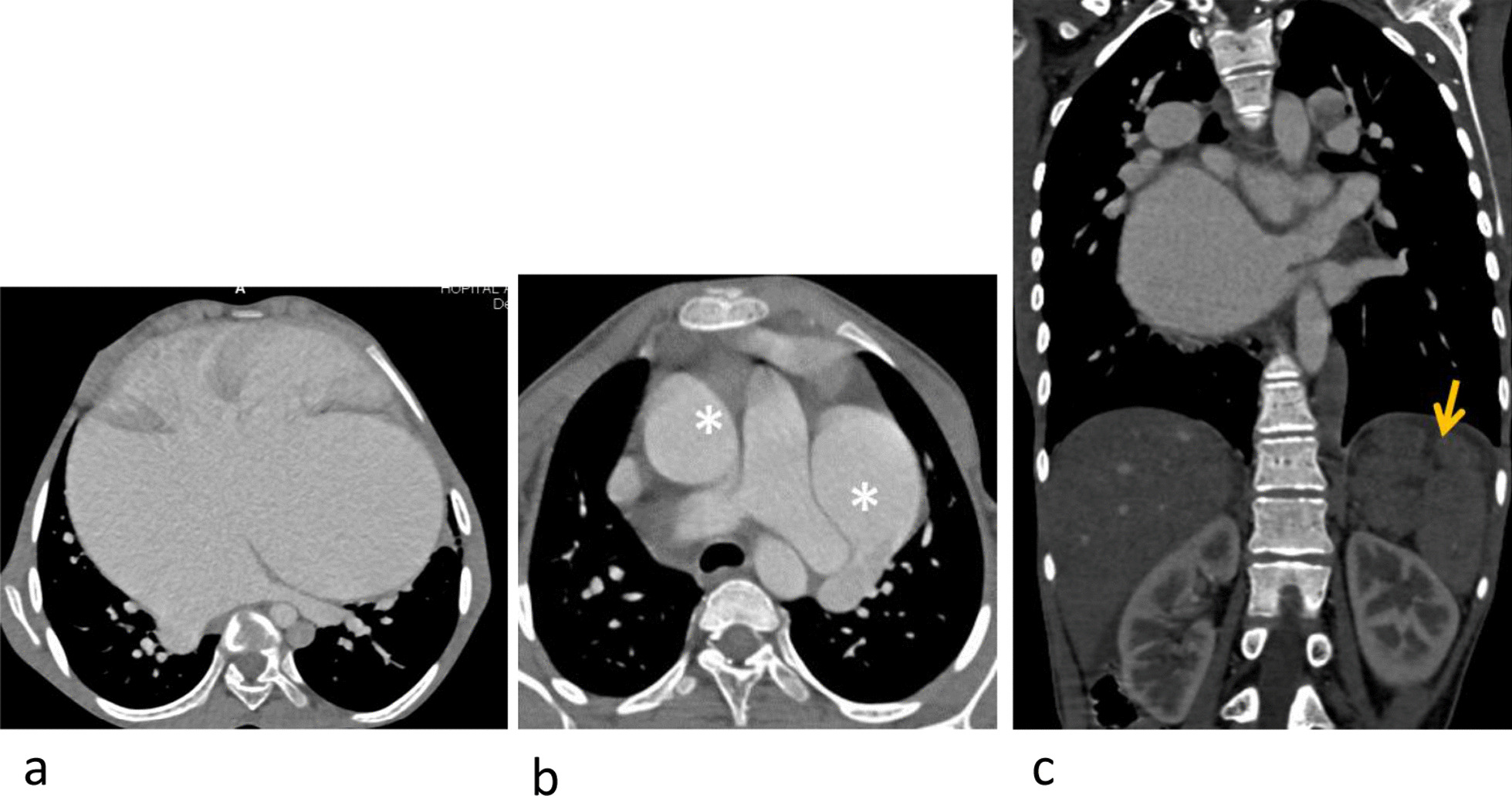
Splenic anomaly. Intra-cardiac total anomalous pulmonary venous return (TAPVR): the 4 pulmonary veins flow directly into the right atrium (**a**), associated with a levo-isomerism and inter-atrial communication (IAC). There is also a double superior vena cava (*b) and a polysplenia [yellow arrow (**c**)]

**Fig. 3:**
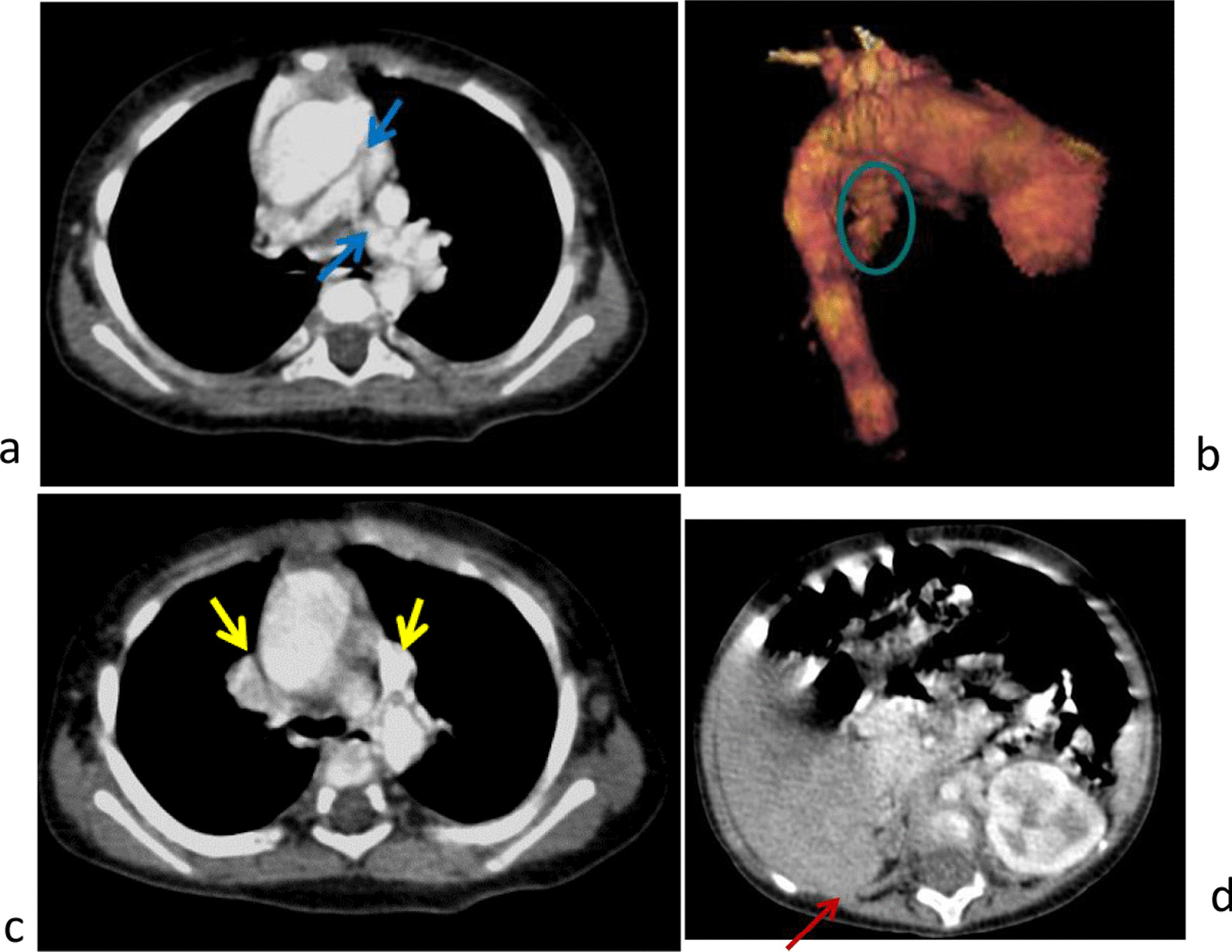
Syndrome del 22q11.2 (Di George). Pulmonary atresia with ventricular septal defect (PAVSD), type II. Stenosis of the root of the pulmonary artery [blue arrows (**a**)]. Patent ductus arteriosus [circle (**b**)] and double superior vena cava [yellow arrows (**c**)]. No visibility of the thymus. Agenesis of the right kidney [red arrow (**d**)]

#### Skeletal anomalies (Table 4; Fig. 4)

Since the CT coverage excluded the limbs, skeletal findings were solely represented by spine anomalies found in 5 patients.

**Fig. 4:**
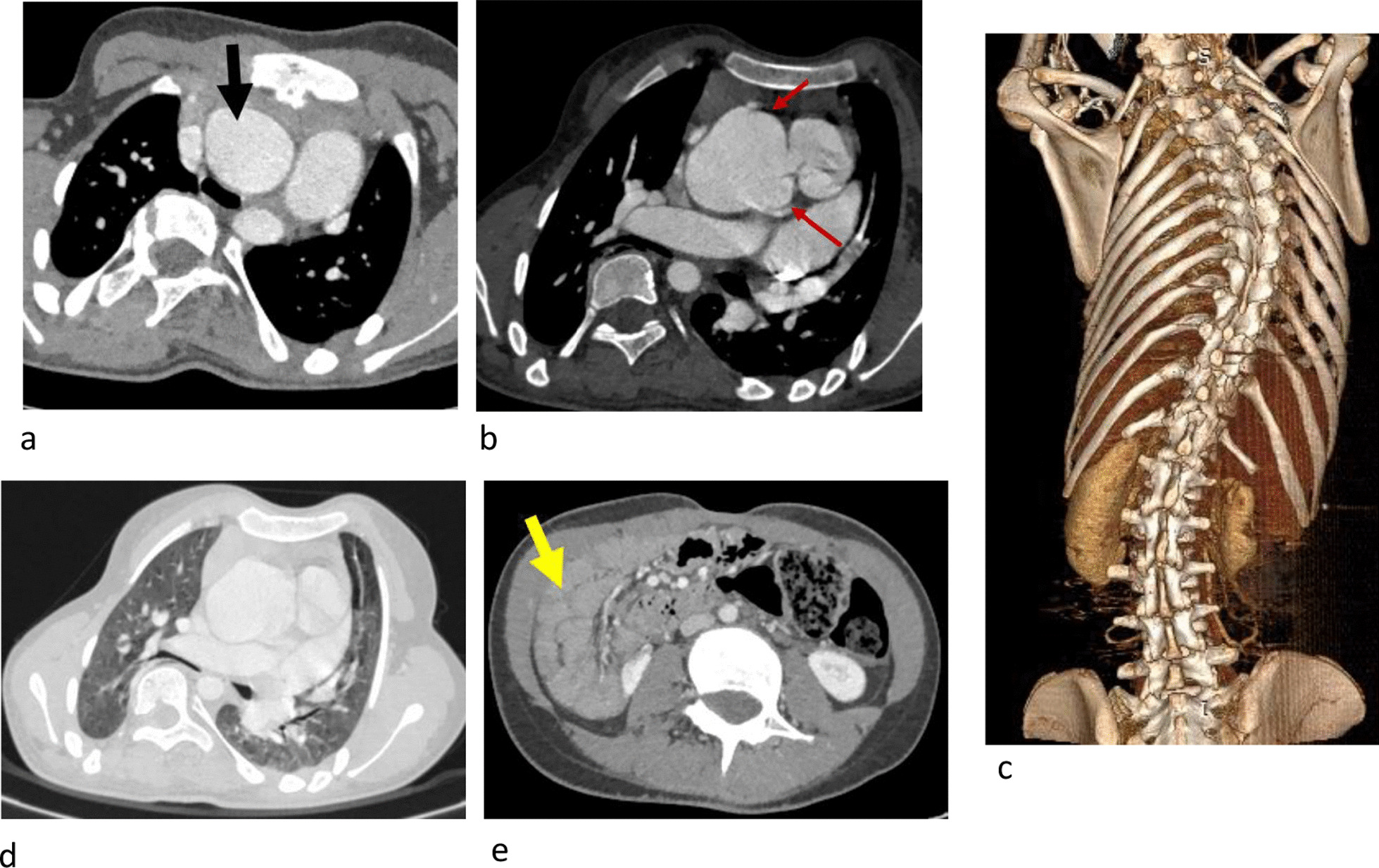
Multiple associated anomalies. 14 years old, Marfan syndrome. Dilation of the aorta [black arrow (**a**)] and Valsalva sinus aneurysmal dilation [red arrows (**b**)]. Dilation of the pulmonary artery. Dorsal scoliosis (**c**) with thoracic deformation and hypoplasia of both lungs (**d**). Presence of a complete common mesentery; the small intestinal loops being located to the right of the colon [yellow arrow (**e**)]

#### Syndromic associations (Table 5; Figs. 5, 6)

A total of 28 cases of syndromic association were identified, representing approximately 16% of the patients presenting a congenital heart disease and approximately 27% of the patients (28 of 105) positive for a non-cardiovascular CT finding. Heterotaxic syndromes were predominant.

**Table 5:** Distribution of syndromic congenital heart diseases

Syndromes	n = Patients
Heterotaxic syndromes	14
Down Syndrome	4
Williams Beuren	3
Kartagener	3
22q11.2 (Di-George)	2
VACTERL	1
Marfan	1

**Fig. 5:**
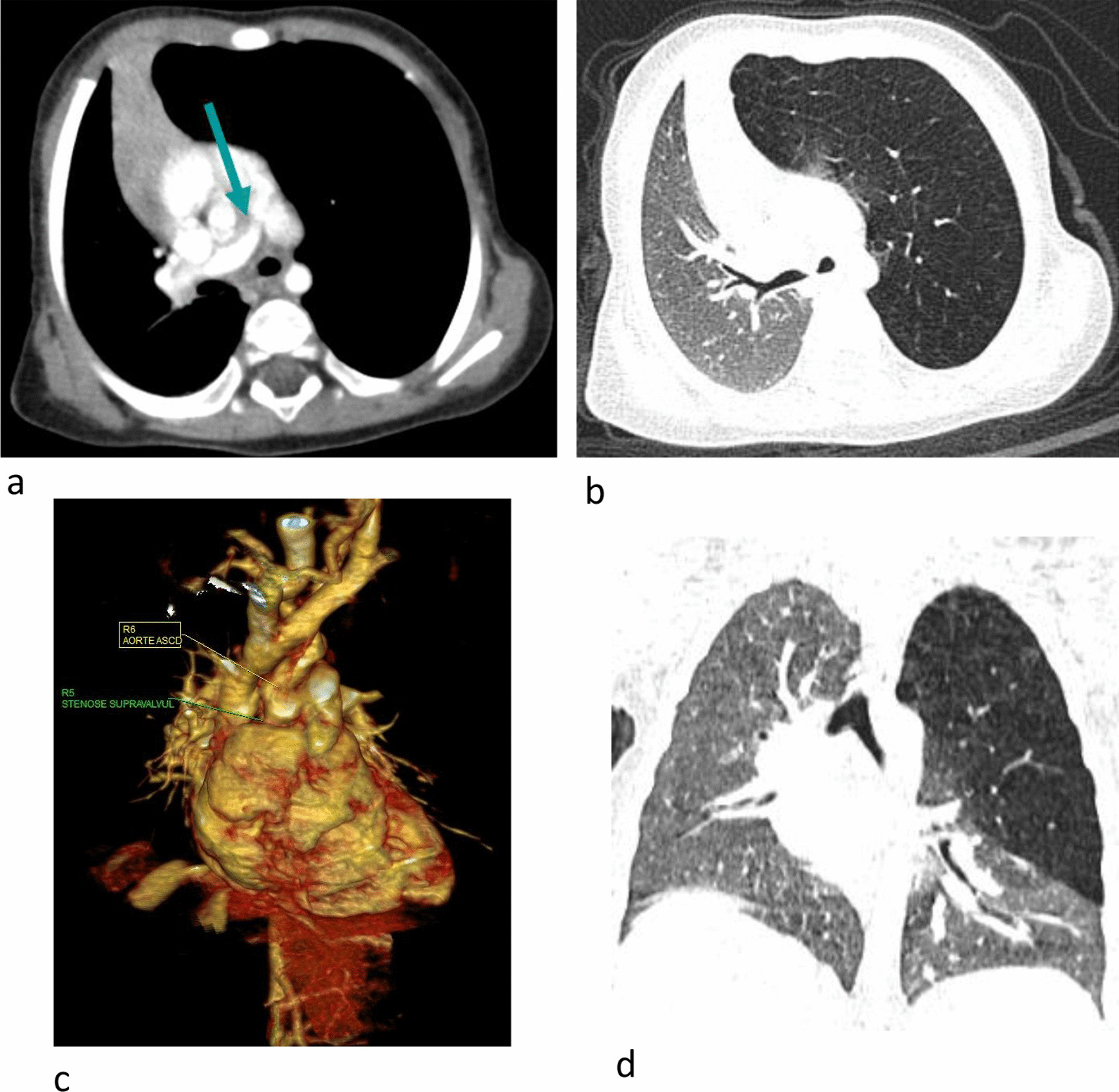
Williams Beuren syndrome. Supravalvular aortic stenosis associated with a tight stenosis of the root of the right pulmonary artery [arrows (**a**, **c**)]. Congenital lobar emphysema of the superior pulmonary left lobe (**b**, **d**).

**Fig. 6:**
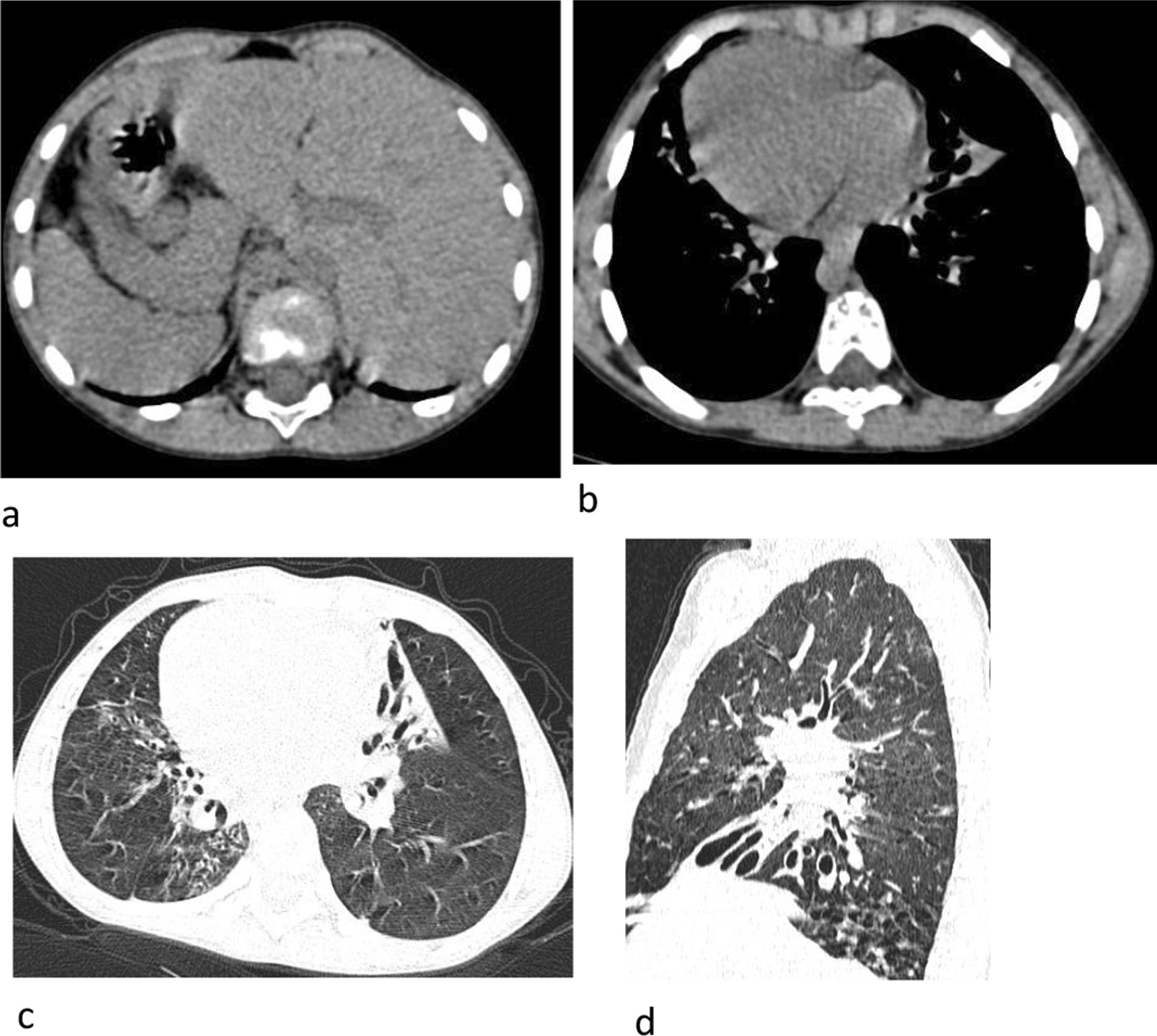
Kartagener syndrome. Situs inversus (**a**, **b**). Bilateral bronchiectasis (**c**, **d**)

## Discussion

The prevalence of non-cardiovascular anomalies is 58% in our study. In similar studies using CT angiography as the exploration technique, this prevalence is 83% in the series by Malik et al. [21] and 31.6% in the series by Rodriguez Martin et al. [20].

There is a moderate variability between these different series. Also, despite the use of a 64 detectors MDCT scanner, Malik et al. [21] and Rodriguez Martin et al. [20] showed a significant difference in the prevalence of the non-cardiovascular findings.

In view of these observations, we deduce a few reflections: we used a 16-detectors MDCT scanner. It is clear that a 64-detectors machine is more efficient. Also, it would be justified to conclude that the higher prevalence of Malik et al. [21], compared to our study, is in a large part, due to the use of a more efficient machine. But this reasoning fails when we compare the results of Malik et al. [21] with those of Rodriguez Martin et al. [20]. This could mean that the performance of the MDCT scanner and the characteristics of the population are both, the key factors determining the prevalence of non-cardiovascular findings. Because the study by Malik et al. [21] was carried out in a reference center specialized in pediatric vascular imaging; therefore, recruiting patients in greater number and severity; whereas our study and that of Rodriguez Martin et al. [20] were carried out in a pediatric imaging department with several activities, some of which are dedicated to pediatric cardiovascular imaging.

Nevertheless, it is obvious that non-cardiovascular findings are frequent. A common point of these three studies is the higher frequency of pulmonary and airway findings observed in 41% our patients, versus 59% in Malik et al. series [21] and 16.7% in that of Rodriguez Martin et al. [20].

Ghadimi Mahani et al. [22] were interested in the prevalence of extra-cardiac abnormalities found on MR angiographies in children with congenital heart disease. They found an overall prevalence of 16.5% of non-cardiac anomalies with a predominance of abdominal findings (51% versus 31% for intrathoracic findings). This difference can be explained by the lower performance of MRI for the study of thoracic structures.

These above-mentioned studies are listed along with ours in (Table 6) showing comparison of their key points.

**Table 6:** Comparative listing of the different studies

Study	Imaging technic	Period	Type	Number of patients (n)	Prevalence of non-cardiovascular findings (%)	Significant non-cardiovascular findings (%)	Predominant findings
Malik et al. [21]	CTA	2 years and 4 months	Retrospective	300	83	73.7	Lungs
Ghadimi Mahani et al. [22]	MRA	4 years and 10 months	Retrospective	740	16.5	2.6	Abdominal
Rodriguez Martin [20]	CTA	5 years	Retrospective	222	31.6	22.7	Lungs
Our study	CTA	4 years and 3 months	Retrospective	180	58	49	Lungs

The importance of these findings varies. Some of them are minor, without significant clinical consequence; others are major, requiring additional investigations or even appropriate immediate treatment. In our series, 49% of patients with congenital heart disease have at least one significant non-cardiac finding, requiring additional investigations or specific immediate or deferred management. This rate was 74% in the series of Malik et al. [21], higher than in ours.

This high prevalence of significant non-cardiovascular findings detected on examinations performed for congenital heart disease, requires the radiologist to be vigilant and attentive when analyzing a chest CT angiography in children. The impact of associated non-cardiovascular lesions on the diagnostic and therapeutic management of the patient can be crucial [19, 21–24].

However, some authors believe that the detection of non-cardiovascular anomalies on cardiac CT angiographies (especially in adults) can be rather a disadvantage, because their detection often leads to additional diagnostic procedures and additional costs without significant improvement of the patient’s condition [25, 26].

Such reasoning goes against the goal of our study and many other similar ones, because it could cultivate the laziness of the radiologists as regards to the systematic analysis of all the volume acquired. We also believe that ignoring or not reporting such anomalies is a mistake and could have a negative impact on the patient’s outcome.

As a reminder, the data of our study shows that approximately one out of two patients with congenital heart disease have at least one significant non-cardiac finding. Moreover, these significant anomalies can be unexpected, urging the radiologists to look at everything and to report the other findings associated with the congenital heart diseases.

The vast majority of the congenital heart diseases are isolated. However, 20–30% are the expression of a genetic abnormality fitting into a syndromic framework. Genetic progress has been made in identifying several associated genes in polymalformative genetic syndromes that combine congenital heart disease and extra-cardiac manifestations [27–30]. The knowledge of the syndromic character of a heart disease requires a systematic investigation of commonly associated extracardiac abnormalities.

In our series, we identified 28 cases of syndromic heart diseases, representing 17% of all our patients and 27% of patients with non-cardiovascular findings. These syndromic heart diseases have a prevalence of 22% in the series of Malik et al. [21] among which half had genetic confirmation only after radiological suspicion. This again underlines the importance of detecting and reporting these findings.

### Critical analysis of the study

The first bias is the retrospective nature of the study. There is an intrinsic incomplete nature of the recruitment of patients and of the data collection because, for example, if a desired data is missing from the report, it is considered to be really absent when it could have been present if the study had been prospective and we had systematically planned to search for particular anomalies.

Also, given the retrospective nature of our study, there are chances of lack of adequate monitoring of the evolution of non-cardiovascular abnormalities in order to effectively weigh their importance in the management of the patient.

The data collected depends on the skills of a single reader. It is obvious that adding a second one would minimize the risk of omission and add more value to our observations. Despite this, we are confident in our excellent diagnostic ability in congenital heart diseases.

The separation between significant and non-significant findings can sometimes be delicate. In addition, a direct attribution of an anatomical abnormality to a clinical manifestation is not obvious because the pathophysiological implications can be complex.

Last but not least, we used a single source 16-detectors MDCT scanner. It obvious that a better machine would have grant us a better quality of the data.

Nevertheless, despite these limitations, our results are usable. This study allowed us to make a real observation of the prevalence, significance and polymorphism of these non-cardiovascular findings associated with congenital heart disease in children explored by CT angiography.

It should lead us to place more emphasis on the systematic analysis of the acquired volume, in order to detect the maximum associated lesions, since they can have major impact in the management of the patients.

## Conclusion

Non-cardiovascular findings are frequent on chest CT angiography in children with congenital heart disease. The data of our series showed that almost 2/3 of these children with congenital heart disease have one or several non-cardiovascular anomalies, of which up to 50% can be significant requiring additional investigations and / or contributing to the patient's symptoms; therefore, requiring immediate or deferred appropriate management. Their detection is essential for the optimal management of these patients.

These observations underline the importance of a systematic analysis of all the acquired volume of chest CT angiographies.

## Data Availability

The datasets used and/or analyzed during the current study are available from the corresponding author on reasonable request.

## Abbreviations

ALARA: As low as reasonably achievable
MRI: Magnetic resonance imaging
CT: Computed tomography
MIP: Maximum intensity projection
MinIP: Minimum intensity projection
VRT: Volume rendering technic
3D: Three-dimensions
MDCT: Multidetector-row computed tomography

## References

[1] Reller MD, Strickland MJ, Riehle-Colarusso T, Mahle WT, Correa A (2008). Prevalence of congenital heart defects in metropolitan Atlanta, 1998–2005. J Pediatr.

[2] Hoffman JI, Kaplan S (2002). The incidence of congenital heart disease. J Am Coll Cardiol.

[3] Botto LD, Correa A, Erickson JD (2001). Racial and temporal variations in the prevalence of heart defects. Pediatrics.

[4] Hellinger JC, Pena A, Poon M, Chan FP, Epelman M (2010). Pediatric computed tomographic angiography: imaging the cardiovascular system gently. Radiol Clin North Am.

[5] Koestenberger M, Friedberg MK, Ravekes W, Nestaas E, Hansmann G (2012). Non-invasive imaging for congenital heart disease: recent innovations in transthoracic echocardiography. J Clin Exp Cardiolog..

[6] Han BK, Lesser JR (2013). CT imaging in congenital heart disease: an approach to imaging and interpreting complex lesions after surgical intervention for tetralogy of Fallot, transposition of the great arteries, and single ventricle heart disease. J Cardiovasc Comput Tomogr.

[7] Kulkarni A, Hsu HH, Ou P, Kutty S (2016). Computed tomography in congenital heart disease: clinical applications and technical considerations. Echocardiography.

[8] Han BK, Overman DM, Grant K, Rosenthal K, Rutten-Ramos S, Cook D, Lesser JR (2013). Non-sedated, free breathing cardiac CT for evaluation of complex congenital heart disease in neonates. J Cardiovasc Comput Tomogr.

[9] Dillman JR, Hernandez RJ (2009). Role of CT in the evaluation of congenital cardiovascular disease in children. AJR Am J Roentgenol.

[10] Goo HW (2011). Cardiac MDCT in children: CT technology overview and interpretation. Radiol Clin North Am.

[11] Greenberg SB, Bhutta S, Braswell L, Chan F (2012). Computed tomography angiography in children with cardiovascular disease: low dose techniques and image quality. Int J Cardiovasc Imaging.

[12] Haller S, Kaiser C, Buser P, Bongartz G, Bremerich J (2006). Coronary artery imaging with contrast-enhanced MDCT: extracardiac findings. AJR Am J Roentgenol.

[13] Onuma Y, Tanabe K, Nakazawa G, Aoki J, Nakajima H, Ibukuro K, Hara K (2006). Noncardiac findings in cardiac imaging with multidetector computed tomography. J Am Coll Cardiol.

[14] Chia PL, Kaw G, Wansaicheong G, Ho KT (2009). Prevalence of non-cardiac findings in a large series of patients undergoing cardiac multi-detector computed tomography scans. Int J Cardiovasc Imaging.

[15] Dewey M, Schnapauff D, Teige F, Hamm B (2007). Non-cardiac findings on coronary computed tomography and magnetic resonance imaging. Eur Radiol.

[16] Sohns C, Sossalla S, Vollmann D (2011). Extracardiacfindings by 64-multidetector computed tomography in patients with symptomatic atrial fibrillation prior to pulmonal vein isolation. Int J Cardiovasc Imaging.

[17] Bendix K, Jensen JM, Poulsen S (2011). Coronary dual source multi detector computed tomography in patients suspected of coronary artery disease: prevalence of incidental extra-cardiac findings. Eur J Radiol.

[18] May CW, Mansfield WT, Landes AB, Moran AM (2012). Prevalence of noncardiac findings in patients undergoing cardiac magnetic resonance imaging. Sci World J.

[19] Buckens CF, Verkooijen HM, Gondrie MJ, Jairam P, Mali WP, van der Graaf Y (2012). Unrequested findings on cardiac computed tomography: looking beyond the heart. PLoS ONE.

[20] J. I. Rodríguez Martín, M. Bret Zurita, E. Cuesta López, L. Polo López, A. Cartón Sánchez, F. Gutiérrez-Larraya Aguado; Madrid/ES. Unexpected findings in cardiac imaging of pediatric patients with congenital heart disease using 64 detector computed tomography. ECR 2019/ C-2538. 10.26044/ecr2019/C-2538

[21] Malik A, Hellinger JC, Servaes S (2017). Prevalence of non-cardiovascular findings on CT angiography in children with congenital heart disease. Pediatr Radiol.

[22] Ghadimi Mahani M, Morani AC, Lu JC (2016). Non-cardiovascular findings in clinical cardiovascular magnetic resonance imaging in children. Pediatr Radiol.

[23] Sohns C, Sossalla S, Vollmann D (2011). Extra cardiac findings by 64-multidetector computed tomography in patients with symptomatic atrial fibrillation prior to pulmonal vein isolation. Int J Cardiovasc Imaging.

[24] Gil BN, Ran K, Tamar G, Shmuell F, Eli A (2007). Prevalence of significant noncardiac findings on coronary multidetector computed tomography angiography in asymptomatic patients. J Comput Assist Tomogr.

[25] Budoff MJ, Fischer H, Gopal A (2006). Incidental findings with cardiac CT evaluation: should we read beyond the heart?. Catheter Cardiovasc Interv.

[26] Budoff MJ, Gopal A (2007). Incidental findings on cardiac computed tomography. Should we look?. J Cardiovasc Comput Tomogr.

[27] Calcagni G, Unolt M, Digilio MC, Baban A, Versacci P, Tartaglia M, Baldini A, Marino B (2017). Congenital heart disease and genetic syndromes: new insights into molecular mechanisms. Expert Rev Mol Diagn.

[28] Ko JM (2015). Genetic Syndromes associated with Congenital Heart Disease. Korean Circ J.

[29] Mani A, Alizadehasl A, Sadeghpour A, Kyavar M, Alizadehasl A (2014). Syndromic congenital heart diseases. Comprehensive approach to adult congenital heart disease.

[30] Fahed AC, Gelb BD, Seidman JG, Seidman CE (2013). Genetics of congenital heart disease: the glass half empty. Circ Res.

